# Association between low-carbohydrate diet score and incidence of type 2 diabetes among Japanese adults: the JACC Study

**DOI:** 10.1017/jns.2022.122

**Published:** 2023-04-14

**Authors:** Akinori Yaegashi, Takashi Kimura, Takumi Hirata, Hiroyasu Iso, Akiko Tamakoshi

**Affiliations:** 1 Graduate School of Medicine, Hokkaido University, North 15, West 7, Kita-ku, Sapporo 060-8638, Japan; 2 Department of Health and Nutrition, Faculty of Human Science, Hokkaido Bunkyo University, 5-196-1, Kogane-chuo, Eniwa 061-1449, Japan; 3 Faculty of Medicine, Hokkaido University, North 15, West 7, Kita-ku, Sapporo 060-8638, Japan; 4 Institute for Clinical and Translational Science, Nara Medical University, 840 Shijo-cho, Kashihara, Nara 634-8522, Japan; 5 National Center for Global Health and Medicine, 1-21-1 Toyama, Shinjuku-ku, Tokyo 162-8655, Japan

**Keywords:** Asia, Diabetes, Epidemiology, Japanese, Low carbohydrate diet

## Abstract

We prospectively examined the association between low-carbohydrate diet (LCD) score and incidence of type 2 diabetes (T2D) in Japanese adults using Japan Collaborative Cohort Study for Evaluation of Cancer Risk (JACC Study) data. A total of 19 084 (7052 men and 12 032 women) Japanese non-diabetic participants aged 40–79 years, who enrolled in the JACC study between 1988 and 1990, were included in our analysis. Dietary intake was evaluated using a validated food-frequency questionnaire. The overall, animal and vegetable LCD scores were calculated by dividing the study participants into eleven categories based on the percentages of energy from carbohydrates, protein and fat. The incidence of T2D was assessed using a self-administered questionnaire. We used multivariable logistic regression analysis to estimate the odds ratios (ORs) and 95 % confidence intervals (CIs) of incident T2D across the quintile of each LCD score, with adjustment for potential confounders. During the 5-year study period, 490 adults (247 men and 243 women) developed T2D. The multivariable-adjusted OR of incident T2D for the highest *v*. lowest quintiles of overall and animal LCD scores, respectively, were 0·64 (95 % CI 0·42, 0·99) and 0·83 (95 % CI 0·55, 1·27) for men, 0·78 (95 % CI 0·51, 1·18) and 0·84 (95 % CI 0·57, 1·24) for women. The vegetable LCD score was associated with a lower risk of T2D in men (OR 0·51; 95 % CI 0·33, 0·77). Our results suggest that diets lower in carbohydrates and higher in fat and protein are unlikely to higher the T2D risk among Japanese individuals.

## Introduction

Diabetes mellitus (DM) is a serious, life-threatening health problem characterised by high blood glucose levels. It is estimated that there will be a 51 % surge in the prevalence of diabetes globally by 2045, from 463 million cases in 2019 to 700 million cases in 2045, with type 2 diabetes (T2D) accounting for approximately 90 % of the total cases of diabetes^(1)^.

Various diets have been reported as strategies for the prevention of diabetes in an umbrella review of meta-analyses^(2)^. Among those studies, an association between low-carbohydrate diet (LCD) scores and the risk of developing diabetes has been reported [adjusted summary hazard ratios with 95 % confidence interval (CI): 1,17 (0·90, 1·51)]^(3)^. The LCD score is a simple summary score created by Halton *et al.*^(4)^ and is calculated by evaluating a diet's relative carbohydrate, protein and fat intake. A higher LCD score indicates a higher intake of protein and fat and a lower intake of carbohydrates, while a lower score indicates the opposite^(4)^.

However, most of the studies included in the meta-analysis examining the association between LCD scores and the development of diabetes were conducted in non-Asian countries^(3)^. Asians, such as the Japanese, have a higher carbohydrate intake than non-Asian populations^(5,6)^. In addition, Japanese and other Asian populations have a high proportion of body fat and abdominal obesity^(7,8)^. These characteristics mean that Asian people have a higher predisposition to insulin resistance at a lesser degree of obesity^(7,8)^. Thus, the results of the aforementioned previous meta-analysis study^(3)^ may not be applicable to Asian countries such as Japan.

Therefore, we prospectively examined the association between LCD score and T2D risk in Japanese adults in Asia using Japan Collaborative Cohort Study for Evaluation of Cancer Risk (JACC Study) data.

## Materials and methods

### Study design and population

The JACC Study was launched in 1988 and 1990 with 110 585 participants aged 40–79 years from 45 Japanese communities. The JACC Study protocol has been reported previously^(9)^. The study protocol was conducted in accordance with the Declaration of Helsinki and was approved by the Ethics Committee of the Faculty of Medicine, Hokkaido University (approval no. 14-044).

**Fig. 1.**
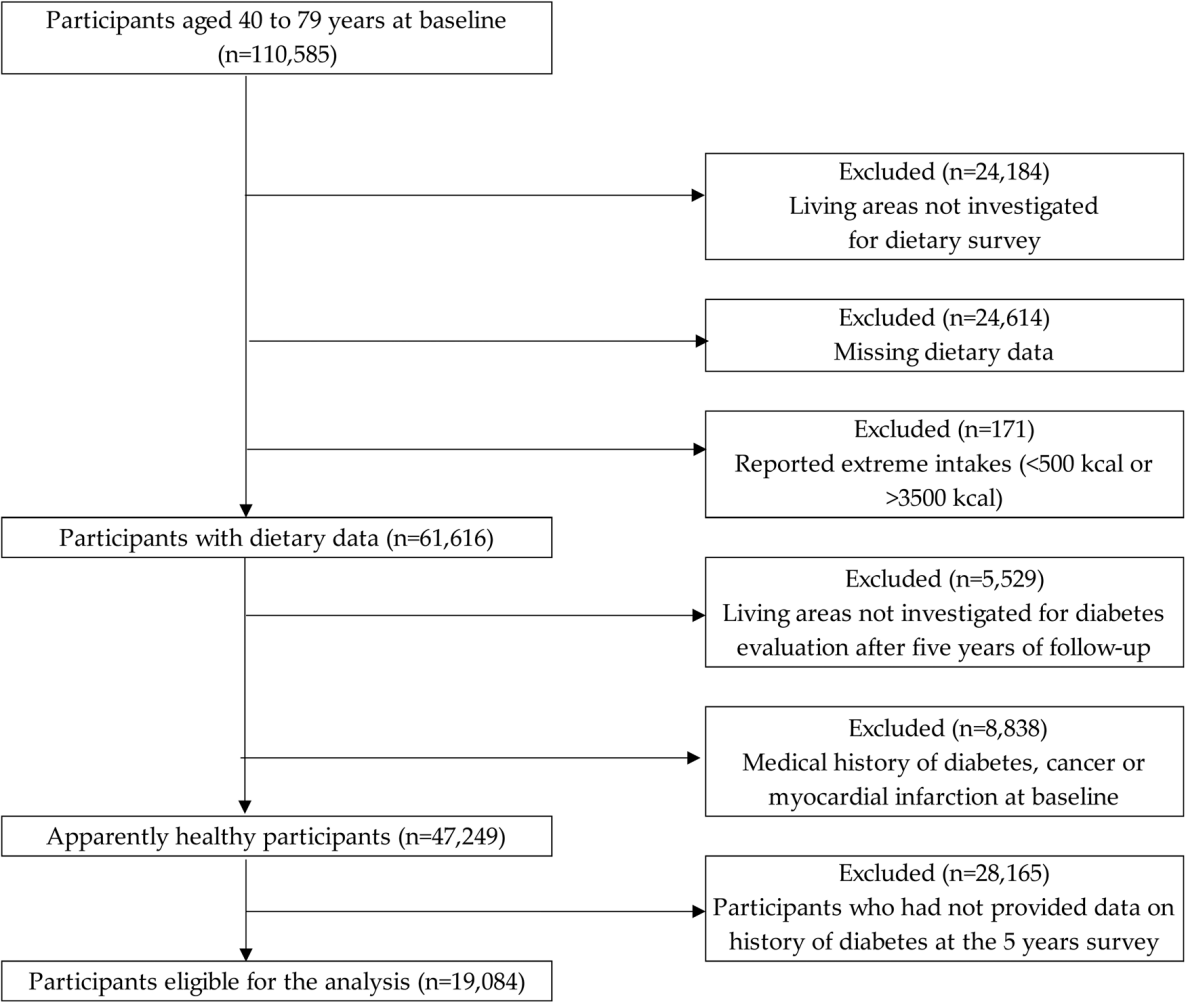
Flow diagram of the study participants.

Participants were excluded according to the following criteria: living areas not investigated for dietary survey (*n* 24 184); missing dietary data (*n* 24 614); reported extreme dietary intakes (<500 kcal or >3500 kcal) (*n* 171); living areas not investigated for diabetes evaluation after 5 years of follow-up (*n* 5529); medical histories of diabetes, cancer or myocardial infarction at baseline (*n* 8838); and participants who had not provided data on history of diabetes at the 5 years survey (*n* 28 165). As a result, 19 084 patients (7052 men and 12 032 women) were ultimately eligible for the present analysis (Fig. 1).

### Dietary assessment

Participants completed their past year's habitual consumption of forty food and drink items in a food-frequency questionnaire (FFQ) that permitted five possible choices of consumption frequencies: rarely, 1–2 times/month, 1–2 times/week, 3–4 times/week and almost every day. Nutrient intakes were computed using the fourth revised version of standard tables of Food Composition in Japan^(10)^. The FFQ-estimated intakes of total protein, animal protein, total fat, animal fat and vegetable fat were correlated with those estimated from the dietary records of the validation study (0·24 for total protein, 0·31 for animal protein, 0·46 for total fat, 0·51 for animal fat and 0·34 for vegetable fat, respectively), as shown previously^(11)^. Carbohydrate (% energy), total protein (% energy), animal protein (% energy), vegetable protein (% energy), total fat (% energy), animal fat (% energy) and vegetable fat (% energy) were calculated as follows by applying Atwater factors.
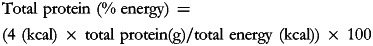

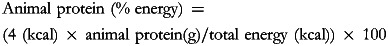

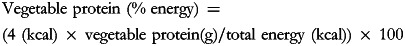

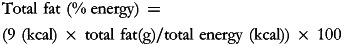

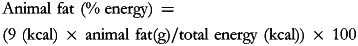

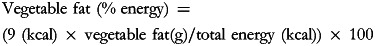

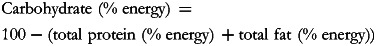


### Calculation of LCD scores

The three LCD scores (overall LCD score, animal LCD score and vegetable LCD score) were calculated accordingly as described in a previous study^(4)^. The overall LCD score was calculated by sorting the study participants into eleven categories based on the percentages of energy from carbohydrates, total protein and total fat, separately for men and women. The carbohydrate categories were scored from 10 to 0 points (i.e. from highest to lowest intake). Conversely, the total protein and total fat categories were scored from 0 to 10 points (i.e. from lowest to highest intake). The three LCD scores ranging from 0 to 30 was derived by summing the carbohydrates, proteins and fats intake points. A higher score indicated a higher proportion of energy intake from total fat and total protein and a lower proportion of energy intake from carbohydrates.

Similarly, the animal LCD score was calculated according to the percentage of energy derived from carbohydrates, animal fat and animal protein. The vegetable LCD score was calculated according to the percentage of energy derived from carbohydrates, vegetable fat and vegetable protein. The criteria for determining the LCD scores are presented in Table 1.

**Table 1.** Criteria for determining the LCD score

Points	Carbohydrate	Total protein	Total fat	Animal protein	Animal fat	Vegetable protein	Vegetable fat
Percentage of energy							
Men							
0	>78·72	<10·03	<11·12	<3·63	<3·48	<5·23	<4·83
1	76·03–78·72	10·03–11·02	11·12–12·79	3·63–4·39	3·48–4·53	5·23–5·80	4·83–5·68
2	73·94–76·02	11·03–11·73	12·80–14·16	4·40–4·92	4·54–5·36	5·81–6·20	5·69–6·30
3	72·30–73·93	11·74–12·29	14·17–15·30	4·93–5·41	5·37–6·08	6·21–6·50	6·31–6·85
4	70·76–72·29	12·30–12·84	15·31–16·31	5·42–5·88	6·09–6·72	6·51–6·77	6·86–7·39
5	69·22–70·75	12·85–13·37	16·32–17·39	5·89–6·39	6·73–7·44	6·78–7·04	7·40–7·89
6	67·56–69·21	13·38–13·95	17·40–18·53	6·40–6·88	7·45–8·17	7·05–7·34	7·90–8·43
7	65·67–67·55	13·96–14·60	18·54–19·85	6·89–7·47	8·18–9·00	7·35–7·66	8·44–9·08
8	63·23–65·66	14·61–15·43	19·86–21·52	7·48–8·23	9·01–10·09	7·67–8·06	9·09–9·85
9	59·61–63·22	15·44–16·75	21·53–23·98	8·24–9·36	10·10–11·82	8·07–8·62	9·86–10·99
10	<59·61	>16·75	>23·98	>9·36	>11·82	>8·62	>10·99
Women							
0	>72·55	<12·56	<14·57	<4·86	<4·75	<6·35	<6·11
1	69·22–72·55	12·56–13·57	14·57–16·91	4·86–5·80	4·75–6·21	6·35–6·76	6·11–7·14
2	66·99–69·21	13·58–14·30	16·92–18·46	5·81–6·47	6·22–7·25	6·75–7·06	7·15–7·93
3	65·23–66·98	14·31–14·89	18·47–19·70	6·48–7·04	7·26–8·08	7·07–7·34	7·94–8·54
4	63·57–65·22	14·90–15·42	19·71–20·83	7·05–7·57	8·09–8·87	7·35–7·61	8·55–9·13
5	62·10–63·56	15·43–15·91	20·84–21·91	7·58–8·08	8·88–9·63	7·62–7·87	9·14–9·67
6	60·63–62·09	15·92–16·46	21·92–22·94	8·09–8·59	9·64–10·40	7·88–8·15	9·68–10·21
7	59·02–60·62	16·47–17·04	22·95–24·11	8·60–9·21	10·41–11·25	8·16–8·45	10·22–10·82
8	57·11–59·01	17·05–17·75	24·12–25·53	9·22–9·95	11·26–12·34	8·46–8·80	10·83–11·52
9	54·05–57·10	17·76–18·81	25·54–27·66	9·96–11·07	12·35–13·84	8·81–9·31	11·53–12·59
10	<54·05	>18·81	>27·66	>11·07	>13·84	>9·31	>12·59

LCD, low carbohydrate diet.

Overall LCD score: This score was calculated by sorting the study participants into eleven categories based on the percentages of energy from carbohydrates, total protein and total fat. Animal LCD score: This score was calculated by sorting the study participants into eleven categories based on the percentage of energy from carbohydrates, animal protein and animal fat. Vegetable LCD score: This score was calculated by sorting the study participants into eleven categories based on the percentage of energy from carbohydrates, vegetable protein and vegetable fat.

### Assessment of diabetic status

Since the exact date of diabetes diagnosis was unknown, the cumulative incidence rate over 5 years was used. Thus, patients with onset diabetes were considered to be those without diabetes at baseline who reported being diagnosed with diabetes by a physician during the 5-year study period. The self-reported diagnosis of diabetes among our participants was validated by comparison with therapy data and laboratory findings. These had 70 and 95 % sensitivity and specificity in men, and 75 and 98 % in women, respectively^(12)^.

### Statistical analysis

Participants were classified into quintiles of the three LCD scores according to sex. Using the lowest quintile of the three LCD scores (overall LCD score, animal LCD score and vegetable LCD score) as a reference, logistic regression analyses were used to estimate the odds ratios (ORs) and 95 % CI of incident T2D. The multivariable model was adjusted for age (continuous); family history of diabetes (yes, no); family history of hypertension (yes, no); smoking status (never, former smoker and current smoker); body mass index (BMI; <18·5, 18·5–24·9, 25·0–30·0 and >30·0 kg/m^2^); walking hours (almost none, daily 0·5, 0·6–0·9 and ≥1·0 h); exercise hours (almost none, weekly 1–2, 3–4 and ≥5 h); alcohol habit (never, former drinker and current drinker) and energy intake (continuous). Tests for trends were conducted using the median value for each quintile of the three LCD scores as a continuous variable. All analyses were conducted separately according to sex because Japanese people have different dietary habits depending on sex (2118 kcal, carbohydrate: 274·6 g, total protein: 77·7 g, total fat: 66·4 g in men; 1709 kcal, carbohydrate: 224·6 g, total protein: 65·7 g, total fat: 56·7 g in women)^(13)^. After stratification by sex, further stratified analyses by other factors, such as BMI, were also considered but were not performed due to the low incidence of diabetes in this study. A significance level for a two-sided *P*-value <0·05 using the SAS 9.4 software (SAS Institute Inc., Cary, NC, USA) was applied for all statistical analyses.

## Results

During the 5-year study period, 490 participants developed T2D (2·6 %): 247 (3·5 %) men and 243 (2·0 %) women.

The characteristics of the study participants by quintile of the LCD scores are shown in Table 2. Men and women with the highest score of the three LCD scores were generally more likely to be current smokers than those with the lowest score. Among men, participants with the highest score of the three LCD scores were older than those with the lowest score. Conversely, in women, participants with highest overall and animal LCD scores were younger than those with lowest scores.

**Table 2.** Baseline characteristics of participants according to quintile categories of the LCD score

	Overall LCD score				Animal LCD score				Vegetable LCD score			
	Q1 (low)		Q5 (high)		Q1 (low)		Q5 (high)		Q1 (low)		Q5 (high)	
	Mean or *n*	sd or %	Mean or *n*	sd or %	Mean or *n*	sd or %	Mean or *n*	sd or %	Mean or *n*	sd or %	Mean or *n*	sd or %
Men												
Median score (range)	2 (0–5)		28 (25–30)		3 (0–6)		28 (25–30)		4 (0–7)		26 (23–30)	
Age, years	54·5	9·3	58·0	9·8	54·9	9·4	57·6	9·9	54·0	9·2	57·6	9·8
Family history of diabetes, *n* (%)	114	8·3	138	10·1	128	8·5	136	10·5	134	8·9	167	11·2
Family history of hypertension, *n* (%)	493	35·9	462	33·8	549	36·4	433	33·5	548	36·6	526	35·3
Current smoker, *n* (%)	798	58·1	590	43·2	844	55·9	588	45·6	891	59·4	634	42·5
Current drinker, *n* (%)	1233	89·7	811	59·3	1303	86·3	820	63·5	1406	93·8	824	55·3
BMI, kg/m^2^	22·7	2·6	22·6	4·5	22·7	2·7	22·6	4·5	22·7	2·6	22·6	2·8
Sports ≥ 5 h/week, *n* (%)	91	6·6	118	8·6	96	6·4	114	8·8	98	6·5	133	8·9
Walking ≥ 0·5 h/week, *n* (%)	1201	87·4	1200	87·7	1316	87·2	1143	88·5	1302	86·9	1302	87·3
Energy intake, kcal/d	1810	471	1434	431	1833	473	1431	429	1754	457	1548	467
Carbohydrate, % energy/d	79·2	2·9	58·8	4·3	78·4	3·4	59·1	4·8	77·4	4·2	61·4	5·5
Total protein, % energy/d	10·0	1·2	16·9	1·7	10·4	1·4	16·6	1·9	10·5	1·6	16·1	1·9
Animal protein, % energy/d	3·8	1·1	9·3	1·8	3·7	0·9	9·5	1·6	4·9	1·7	7·8	2·1
Vegetable protein, % energy/d	6·2	1·2	7·6	1·3	6·7	1·2	7·1	1·3	5·6	0·9	8·3	1·0
Total fat, % energy/d	10·9	2·0	24·4	3·1	11·2	2·2	24·4	3·3	12·1	2·9	22·5	3·9
Animal fat, % energy/d	4·1	1·7	11·1	2·9	3·8	1·3	11·9	2·4	5·4	2·4	9·3	3·0
Vegetable fat, % energy/d	5·4	1·4	10·4	2·2	6·0	1·7	9·7	2·4	5·0	1·1	10·9	1·6
SFA, % energy/d	3·2	0·8	7·2	1·5	3·2	0·7	7·5	1·4	3·8	1·3	6·3	1·5
MUFA, % energy/d	3·2	0·7	7·6	1·2	3·3	0·7	7·7	1·1	3·7	1·0	6·8	1·4
PUFA, % energy/d	3·0	0·7	6·0	1·1	3·3	0·9	5·7	1·3	3·0	0·6	6·1	1·0
Women												
Median score (range)	2 (0–5)		28 (25–30)		3 (0–6)		27 (24–30)		4 (0–7)		25 (23–30)	
Age, years	56·9	9·7	55·0	9·2	57·3	9·6	54·4	9·2	56·0	9·7	56·2	9·3
Family history of diabetes, *n* (%)	230	10·0	317	10·4	248	9·8	268	11·1	263	11·3	211	9·7
Family history of hypertension, *n* (%)	767	33·4	986	36·6	830	32·7	910	37·6	808	34·7	764	35·2
Current smoker, *n* (%)	139	6·1	56	2·5	136	5·4	72	3·0	152	6·5	44	2·0
Current drinker, *n* (%)	640	27·9	502	22·5	636	25·1	607	25·1	775	33·3	371	17·1
BMI, kg/m^2^	23·0	6·5	22·7	2·9	22·9	3·1	22·6	2·9	23·0	6·4	22·9	3·0
Sports ≥ 5 h/week, *n* (%)	101	4·4	117	5·3	121	4·8	123	5·1	103	4·4	108	5·0
Walking ≥ 0·5 h/week, *n* (%)	1999	87·1	1928	86·5	2217	87·4	2111	87·1	2061	88·4	1861	85·8
Energy intake, kcal/d	1380	380	1302	370	1381	382	1303	360	1331	362	1340	345
Carbohydrate, % energy/d	73·2	3·6	53·2	3·5	72·3	4·2	54·0	4·0	71·1	5·1	56·1	4·7
Total protein, % energy/d	12·5	1·3	18·9	1·6	12·9	1·6	18·4	1·8	13·1	1·8	18·0	1·9
Animal protein, % energy/d	5·0	1·4	11·0	1·8	5·0	1·2	11·0	1·7	6·4	2·0	9·1	2·0
Vegetable protein, % energy/d	7·5	1·1	7·9	1·3	8·0	1·2	7·5	1·1	6·7	0·7	8·9	0·9
Total fat, % energy/d	14·3	2·6	27·9	2·7	14·8	2·9	27·5	2·8	15·8	3·6	25·9	3·3
Animal fat, % energy/d	5·7	2·2	12·9	3·0	5·2	1·8	13·6	2·4	7·4	3·0	10·4	2·9
Vegetable fat, % energy/d	7·0	1·8	11·4	2·2	7·8	2·3	10·6	2·3	6·2	1·2	12·5	1·6
SFA, % energy/d	4·3	1·1	8·2	1·4	4·2	0·9	8·4	1·2	5·1	1·5	7·1	1·4
MUFA, % energy/d	4·3	0·9	8·7	1·1	4·3	0·9	8·7	1·1	4·9	1·3	7·8	1·3
PUFA, % energy/d	3·8	0·9	6·7	1·1	4·2	1·1	6·2	1·2	3·6	0·7	6·9	0·9

LCD, low-carbohydrate diet; Q, quintile; BMI, body mass index; SFA, saturated fatty acid; MUFA, monounsaturated fatty acid; PUFA, polyunsaturated fatty acid.

Data are mean ± standard deviation unless otherwise indicated.

ORs and 95 % CIs of T2D according to the three LCD scores in men and women are shown in Table 3. No evidence of an association of overall, animal LCD scores and a higher risk of T2D after adjusting for covariates in both sexes. The vegetable LCD score was significantly associated with a lower risk of T2D (*P* for trend = 0·004); the multivariable-adjusted OR (95 % CI) for the highest quintile of the score was 0·51 (0·33–0·77) compared with the lowest quintile in men, but this was not the case in women.

**Table 3.** Odds ratios and 95 % confidence intervals of type 2 diabetes according to quintile categories of the LCD score

	Q1 (low)	Q2	Q3	Q4	Q5 (high)	*P* for trend
Men						
Overall LCD score						
Median score (range)	2 (0–5)	8 (6–11)	15 (12–18)	22 (19–24)	28 (25–30)	
No. of participants	1375	1333	1607	1371	1367	
No. of cases	62	35	55	52	43	
Age adjusted model	1·00 (reference)	0·57 (0·37, 0·86)	0·73 (0·51, 1·06)	0·79 (0·54, 1·15)	0·62 (0·42, 0·92)	0·139
Multivariable model	1·00 (reference)	0·58 (0·38, 0·89)	0·75 (0·51, 1·09)	0·80 (0·54, 1·18)	0·64 (0·42, 0·99)	0·213
Animal LCD score						
Median score (range)	3 (0–6)	10 (7–12)	15 (13–17)	21 (18–24)	28 (25–30)	
No. of participants	1510	1411	1230	1610	1291	
No. of cases	59	38	50	55	45	
Age adjusted model	1·00 (reference)	0·68 (0·45, 1·04)	1·02 (0·70, 1·50)	0·84 (0·58, 1·22)	0·82 (0·55, 1·23)	0·538
Multivariable model	1·00 (reference)	0·68 (0·45, 1·04)	1·03 (0·70, 1·52)	0·84 (0·57, 1·23)	0·83 (0·55, 1·27)	0·591
Vegetable LCD score						
Median score (range)	4 (0–7)	10 (8–12)	15 (13–17)	20 (18–22)	26 (23–30)	
No. of participants	1499	1313	1443	1306	1491	
No. of cases	75	39	44	47	42	
Age adjusted model	1·00 (reference)	0·56 (0·38, 0·83)	0·57 (0·39, 0·83)	0·65 (0·45, 0·95)	0·49 (0·33, 0·72)	0·001
Multivariable model	1·00 (reference)	0·56 (0·38, 0·84)	0·59 (0·40, 0·88)	0·66 (0·45, 0·97)	0·51 (0·33, 0·77)	0·004
Women						
Overall LCD score						
Median score (range)	2 (0–5)	9 (6–12)	16 (13–18)	21 (19–24)	28 (25–30)	
No. of participants	2296	2670	2389	2449	2228	
No. of cases	57	54	45	48	39	
Age adjusted model	1·00 (reference)	0·84 (0·58, 1·23)	0·79 (0·53, 1·18)	0·82 (0·56, 1·21)	0·74 (0·49, 1·11)	0·159
Multivariable model	1·00 (reference)	0·83 (0·57, 1·22)	0·85 (0·57, 1·27)	0·90 (0·60, 1·33)	0·78 (0·51, 1·18)	0·348
Animal LCD score						
Median score (range)	3 (0–6)	10 (7–12)	15 (13–17)	20 (18–23)	27 (24–30)	
No. of participants	2536	2309	2165	2599	2423	
No. of cases	65	45	43	44	46	
Age adjusted model	1·00 (reference)	0·79 (0·54, 1·16)	0·82 (0·55, 1·21)	0·69 (0·47, 1·02)	0·80 (0·54, 1·17)	0·155
Multivariable model	1·00 (reference)	0·80 (0·54, 1·18)	0·86 (0·58, 1·28)	0·76 (0·52, 1·13)	0·84 (0·57, 1·24)	0·325
Vegetable LCD score						
Median score (range)	4 (0–7)	11 (8–13)	16 (14–17)	20 (18–22)	25 (23–30)	
No. of participants	2331	2703	2206	2622	2170	
No. of cases	60	50	35	56	42	
Age adjusted model	1·00 (reference)	0·74 (0·50, 1·08)	0·63 (0·41, 0·95)	0·84 (0·58, 1·21)	0·74 (0·50, 1·11)	0·207
Multivariable model	1·00 (reference)	0·80 (0·54, 1·17)	0·69 (0·45, 1·06)	0·92 (0·63, 1·35)	0·83 (0·55, 1·24)	0·476

LCD, low-carbohydrate diet; Q, quintile. The multivariable model was adjusted for age (continuous); family history of diabetes (yes, no); family history of hypertension (yes, no); smoking status (never, former smoker and current smoker); body mass index (<18.5, 18.5–24.9, 25.0–30.0 and >30.0 kg/m^2^); walking hours (almost none, daily 0.5, 0.6–0.9 and ≥1.0 h); exercise hours (almost none, weekly 1–2, 3–4 and ≥5 h); alcohol habit (never, former drinker and current drinker) and energy intake (continuous).

## Discussion

In the present study, overall and animal LCD scores were not associated with the risk of T2D in both sexes. The vegetable LCD score was associated with a lower risk of T2D in men but not in women.

Previous studies reported that a higher animal LCD score is associated with a higher incidence of diabetes^(14,15)^. In contrast to the findings of those studies^(14,15)^, our results showed that a higher animal LCD score was not associated with a higher incidence of diabetes. This discrepancy may be due to the difference in fish and meat intake between Japanese individuals in the present study and Americans in the previous study^(14,15)^ (fish: Japan 48·60 kg/capita/year, United States 21·51 kg/capita/year; meat: Japan 49·45 kg/capita/year, United States 115·13 kg/capita/year)^(16)^. A previous study reported that intake of long-chain *n*-3 fatty acids, such as fish, has been hypothesised to have beneficial effects on insulin resistance and T2D because of these fatty acids’ ability to inhibit inflammatory pathways and to suppress the expression of genes related to lipid metabolism^(17)^. Additionally, iron, which is present in red meat, increases oxidative stress. This can damage pancreatic beta cells^(18)^. Thus, among populations with high fish and low meat intakes, such as the Japanese, it is likely that a high animal LCD score may not lead to a higher risk for diabetes. Our results that the animal LCD score did not lead to a higher T2D risk is consistent with the findings of Nanri *et al.* in Japan^(19)^.

The present study showed that a higher vegetable LCD score was associated with a lower incidence of T2D in men. This can be attributed to the high vegetable fat intake. Some studies have reported a lower risk of T2D associated with a high vegetable fat intake^(20,21)^. Additionally, in a meta-analysis^(22)^, alpha-linolenic acid, which is abundant in certain plants, has been associated with a lower risk of T2D.

In the present study, no evidence of an association of was found between the three LCD scores and a higher risk of T2D in both sexes. In this regard, our results disagree with those of previous studies^(14,15)^. This might be due to differences in the insulin secretory capacity and % energy from carbohydrates between this Japanese study and the previous studies conducted in the United States^(14,15)^. Japanese individuals and other Asians have a more impaired insulin secretion function^(20)^, and the percentage of energy that they get from carbohydrates is higher than that of non-Asians^(5,6)^. Thus, regardless of the type of protein or fat intake, high LCD scores (i.e. lower carbohydrate and higher protein and fat intake) in Japanese individuals may not have a higher risk of T2D. On the other hand, the differences in results between our study in Japan and the previous studies conducted in the United States^(14,15)^ could also be due to the study design. A dietary survey was conducted only at baseline in our Japanese study, and once every 4 years in a previous study in the United States^(14,15)^. Additionally, the follow-up period was 5 years in the present study and 20 years in the previous studies^(14,15)^. Therefore, further long follow-up cohort studies in Japan are required to confirm our results.

Our results indicated that the vegetable LCD score was associated with T2D risk reduction among men but not women. We considered two possibilities for this sex difference. First, this may be partly explained by differences in T2D incidence between men and women. In the present study, 490 participants developed T2D (2·6 %): 247 (3·5 %) men and 243 (2·0 %) women. Second, this might be due to differences in sex hormones^(23)^. Premenopausal women are less prone to T2D than men, which is partly explained by differences in sex steroid hormones^(23)^. Endogenous oestrogen plays a protective role in diverse metabolic regulations including insulin secretion and sensitivity^(23)^. Third, in general, women were more likely to follow a healthy lifestyle in general, and their diet thus may not have made a large difference in the development of diabetes^(24)^.

The present study had several limitations. First, we used self-reported diabetes data, which may have led to misinterpretation. However, previous studies involving the same cohort reported that self-reported diabetes has moderate sensitivity and high specificity when assessing diabetes based on plasma glucose levels and treatment with hypoglycaemic agents^(12)^. Second, dietary intake was examined using self-reporting. The FFQ-estimated intake of nutrients is underestimated according to a validation study^(11)^. Third, this study did not include data on dietary supplement intake. However, our baseline survey was conducted in late 1980, and the Japanese were not overly familiar with dietary supplements at that time, so we believe that this will have little impact on our results. Fourth, the possibility of unmeasured or residual confounding factors cannot be ruled out. Fifth, the three LCD scores described in this study are derived from the relative energy ratio within this population. Even the participants with the lowest carbohydrate-to-energy ratio, i.e. those with the highest LCD score in this population, had a higher carbohydrate-to-energy ratio compared with the American Academy of Family Physicians definition of less than 20 %^(25)^ (58·7 ± 4·3 % for men and 53·2 ± 3·5 % for women); thus, caution should be exercised in interpreting the results. Finally, since this study was conducted in the late 1980s, and the foods and nutrients consumed in 1990 and 2020 are different (1990 *v*. 2020; energy 2026 kcal *v*. 1903 kcal, carbohydrate: 59·2 % energy *v*. 56·3 % energy, protein: 15·5 % energy *v*. 15·1 % energy, fat: 25·3 % energy *v*. 28·6 % energy)^(16)^. Therefore, this study may not be applicable to the Japanese population in 2022.

## Conclusions

Overall, the animal LCD scores were associated with the incidence of T2D in both sexes, while the vegetable LCD score was associated with a lower incidence in men. Our results suggest that, in populations with high fish and low meat intakes, such as the Japanese, diets lower in carbohydrate and higher in fat and protein are not likely to contribute to a higher T2D risk. Further long-term follow-up cohort studies in Japan are required to confirm our results.
